# Hydrocarbon Lubricants Can Control Hydrogen Embrittlement

**DOI:** 10.1038/s41598-020-58294-y

**Published:** 2020-01-28

**Authors:** Monica Ratoi, Hiroyoshi Tanaka, Brian G. Mellor, Joichi Sugimura

**Affiliations:** 1 Faculty of Engineering and Environment, University of Southampton, Southampton, United Kingdom; 2 Research Centre for Hydrogen Industrial Use and Storage, Kyushu University, 744 Motooka, Nishi-ku, Fukuoka, 819-0395 Japan; 3 International Institute for Carbon-Neutral Energy Research, Kyushu University, 744 Motooka, Nishi-ku, Fukuoka, 819-0395 Japan

**Keywords:** Physical chemistry, Mechanical engineering, Materials for energy and catalysis

## Abstract

While it is well known that during RCF tests the formation of nascent catalytic sites on the wear track can break down hydrocarbon molecules to release atomic hydrogen, the potential of the hydrogen environment in fuel cells to hydrocrack the hydrocarbon lubricant in high pressure rolling contacts has so far been ignored. Here we investigate for the first time the ability of the hydrogen environment to generate a chemical tribofilm on the wear track most likely through lubricant hydrocracking, as compared with argon and air environments. Despite the ability of the hydrogen environment to generate a notably larger amount of atomic hydrogen, the chemical tribofilm significantly prevents the formation of atomic hydrogen and its subsequent diffusion through the lattice of steel rolling element bearings. This is of great importance in the lubrication of hydrogen technology and the prevention of Hydrogen embrittlement (HE). An investigation into the prospects of high energy micro-computed-tomography (Micro-CT) as a non-destructive technique for sub-surface damage characterisation in RCF was comparatively performed alongside traditional sectioning methods.

## Introduction

One of the most promising solutions to reducing carbon emissions is to use hydrogen as the fuel as its combustion products are water. However, the use of hydrogen is not without its problems which are principally related to its small atomic size which enables it to diffuse rapidly through the lattice of solid materials and cause hydrogen embrittlement in high strength steels, such as those used in typical lubricated rolling element bearings, which are subject to high service stresses^1–6^. Indeed, previous studies have shown that the reduction in service life caused by hydrogen diffusion is directly proportional to the amount absorbed^4,7–9^ and thus more problems might be anticipated in a hydrogen environment. Atomic hydrogen can originate from various sources such as the environment (i.e. hydrogen gas in fuel cells, water vapour in air) or from decomposition of lubricant (hydrocarbon-based oil and water molecules) on nascent metal surfaces generated by wear. In the high-stress, high-temperature conditions encountered in systems for supplying hydrogen such as compressors, both hydrogen gas and lubricant molecules (oil and solubilized water) can decompose on the fresh metal sites generated on the wear track and create atomic hydrogen^2,5,10–15^. Severe lubrication conditions may produce more activated sites and increase hydrogen generation and diffusion in steel.

However, when optimally formulated or used in specific operating conditions, lubricants can also play an important role in preventing atomic hydrogen damage to the lubricated steel parts and thus extend the service life of materials. Their ability to form a protective film (tribofilm) on the wear track can stop hydrogen damage through preventing atomic hydrogen formation on the nascent steel surfaces and by acting as a barrier to hydrogen permeation.

Previous research has shown that the use of additives which lead to the formation of a tribofilm on the wear track can reduce hydrogen damage. The most studied additives have been the conventional antiwear zinc dialkyldithiophosphate (ZDDP) and extreme pressure trioctyl phosphate (TCP) because of their ability to dynamically generate thick and uniform chemical tribofilms which impede hydrogen generation and permeation while simultaneously preventing excessive wear and seizure in the EHD contacts operating in severe conditions (high pressure and temperature)^1,10,11,16–18^. The novel 2H-WS_2_ nanoadditized lubricants were shown to achieve a 30% reduction of hydrogen permeation in bearings compared to the PAO base oil, while also controlling wear and generating smoother wear tracks^19^.

Another approach has been to use steels treated with black oxide. This generates soft, thin passivating iron oxide tribofilms that offer corrosion protection, some wear resistance and reduce hydrogen embrittlement until the layer is gradually removed by the contact friction^20,21^.

However, no studies have been conducted to ascertain the effect of the environment on the ability of the hydrocarbon lubricant to generate a tribofilm which impedes hydrogen generation and permeation.

The aim of this work was to elucidate the role of lubricant in preventing wear and failure in different environments. The research focused on the effects of the environment: Air, inert Argon (Ar) and Hydrogen (H_2_) on the ability of the lubricant to generate tribofilms on the wear track and reduce hydrogen content, subsurface damage and wear of the bearing specimens.

High pressure rolling contact fatigue tests were conducted on bearing steel in controlled atmospheres lubricated with a synthetic Polyaphaolefin (PAO32) oil. Samples were tested until significant wear or failure was apparent and immediate Thermal Desorption Spectroscopy (TDS) analysis was undertaken for hydrogen content evaluation. Wear loss was analysed using optical profilometry and the wear track chemistry was determined by XPS analysis.

Sub-surface analysis was performed using traditional sectioning methods alongside a comparative investigation into the prospects of high energy micro-computed-tomography (Micro-CT/µ-CT) as a non-destructive technique for sub-surface damage characterisation.

The use of high energy micro-CT to scan bearings as a non-destructive technique to understand surface damage in bearing steel is a recent step in science. The benefit of using CT is the prospect of 360° analysis of pore, crack and inclusion networks with the bonus of scanned specimens remaining intact for further analysis (albeit the dimensions of the specimens for micro-CT scanning are extremely limited), an impossible task for traditional sectioning and etching analysis.

## Materials and Methods

The lubricant used was a synthetic polyalphaolefin base oil, PAO32 (supplied by Idemitsu Kosan Co. Ltd.) with a density of 0.826 g·cm^−3^ and a viscosity as shown in Table 1.

**Table 1:** RCF test conditions.

Temperature	120 °C
Hertzian pressure	4.8 GPa
Entrainment speed	1500 rpm (3.4 m/s)
Initial composite surface roughness	11 nm
Film parameter Λ	2
Test gas	Air/Argon/H_2_
Lubricant	PAO32 28.8 mm^2^/s (40 °C), 5.6 mm^2^/s (100 °C)
Specimens	JIS SUJ2 steel balls (R_q_ 10 nm)/discs (R_q_ 5 nm)

**Figure 1:**
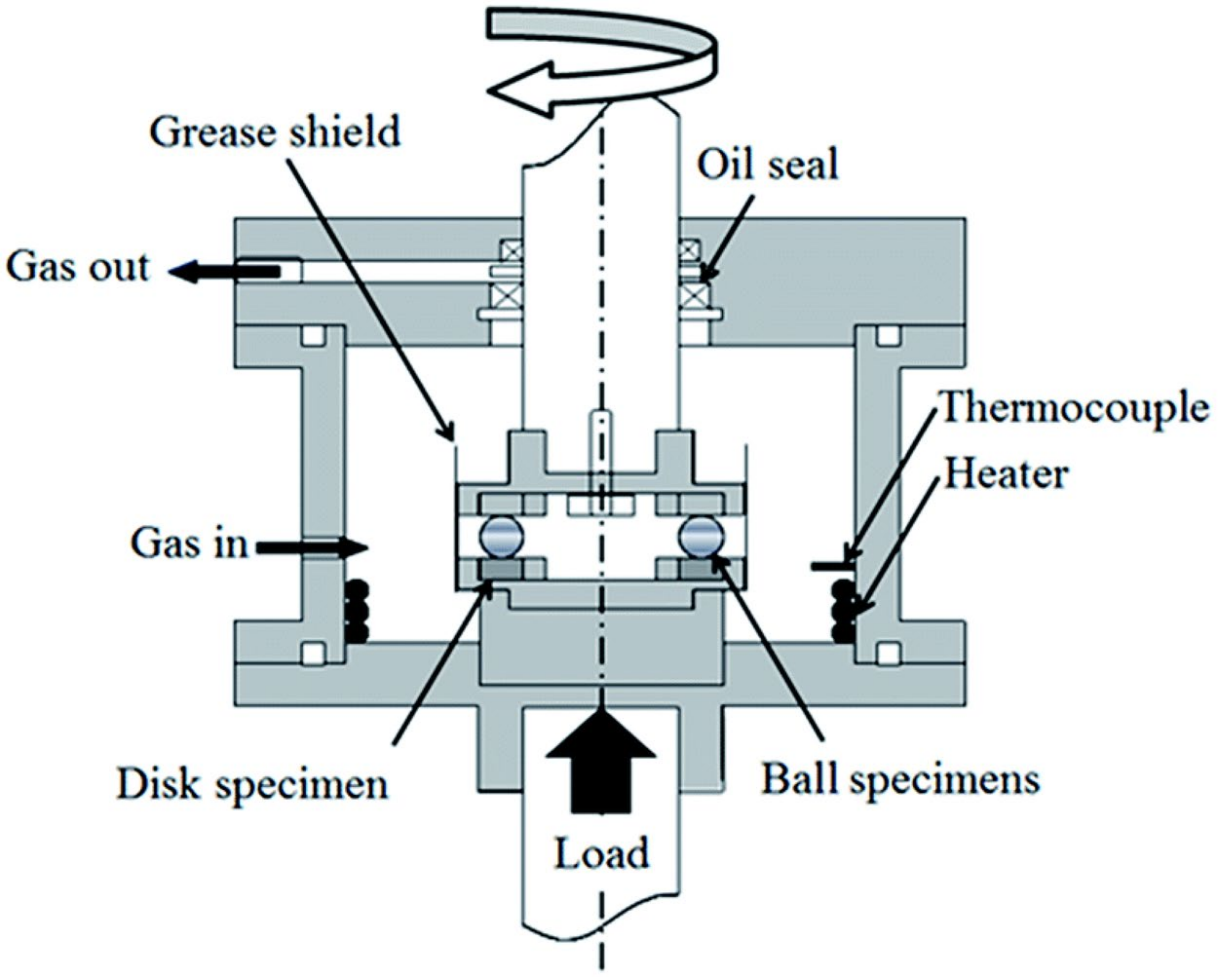
RCF tribometer.

Tribological tests were performed in a ball-on-disc setup test rig. Figure 1 shows a schematic illustration of the apparatus and Table 1 summarizes the test conditions. Thrust bearings were modified to employ the grooved top ring as a guide ring for the ball movement while the lower ring was reversed and therefore had a flat surface in contact with the balls. The ball and disc steel is JIS SUJ2, equivalent to AISI 52100. An R_q_ (root mean square roughness) of 5 nm was achieved for the disc surface by grinding with silicon carbide paper followed by buff polishing with a 3-μm diamond slurry. Only six balls (6.35 mm diameter) were used and were separated by a retainer. The specimens were ultrasonically cleaned (with hexane and acetone) prior to the tribological testing.

A normal load of 2650 N (equivalent to a Hertzian pressure of 4.8 GPa between balls and flat disc and approximately 2.6 GPa between groove and balls) is applied by a lever and the disc/ball contact undergoes rolling through the rotation of the upper shaft. This contact pressure is higher than the calculated pressure encountered during standard operation in bearings and it follows the recommendations from other publications^2,5,7,8,14,15,22^ aiming to accelerate wear, the formation of hydrogen and initiation of embrittlement. The Hertzian pressure employed of 4.8 GPa is almost equal to the shakedown limit (the maximum contact pressure which the material can elastically support in steady state conditions). Theoretically, this implies that plastic deformation below the surface may continue to occur but in practice the pressure reduces due to the change in surface geometry which becomes more conformal through initial deformation and wear, and virtually elastic contact occurs in the rest of the test. The Hertzian contact pressure for the dynamic load rating (load for a rating life of 10^6^ load cycles) of this steel is 4.2 GPa and the contact works in mixed lubrication regime.

The tribometer was equipped with a vibration sensor mounted on the loading lever. Amplitudes detected above a predetermined level (chosen to be that produced by the first flaking event) stopped the test automatically. The fatigue lives were calculated from the number of cycles before the test stopped.

The RCF test were run in three environments: Hydrogen gas (99.97% purity), Argon (99.9995%) and Air (contained approximately 3000 ppm water).

The results from seven tests performed in each environment and the corresponding Weibull plots were reported in two previous studies^7,8^. The current study focused on the analysis of the wear track surface and subsurface of one specimen from each environment which were chosen to have a similar order of flaking lives. The intention was to explore the relationship between hydrogen concentration in steel, wear track damage and the microstructural changes.

TDS (Denshi-Kagaku TDS1200) was used to measure the hydrogen content in the bearing steel specimens (balls and discs). Immediately after the rolling contact tests, the disc and ball specimens were cooled to ambient temperature, cleaned with hexane and acetone in an ultrasonic bath and the disc cut into small pieces (7 × 3.5 × 1 mm, weighing approximately 0.2 g). During the TDS analysis, the cut disc pieces and balls were heated from ambient temperature to 800 °C at a rate of 60 °C/min and 10 °C/min respectively. This leads to the desorption of all gaseous species from the test specimens which are then analysed by a quadrupole mass spectrometer that can measure and analyse the hydrogen species released. Due to its bigger mass and subsequent thermal inertia, a lower rate of heating was used for the ball.

Wear scars were investigated under an optical microscope, Olympus BX41M-LED, to characterise surface defects, deposits and corrosion damage. Wear track profiles were analysed using 3 D non-contact optical profilometry (Alicona Infinite Focus) and validated physically with a contact profilometer apparatus (Talysurf). Optical profilometry was employed to investigate the size (depth and width) of the discs wear track after the RCF test. The 3D surface profile of the wear track was analysed with the supplied software. Multiple wear track profiles were measured in four sections of the disc and used to calculate average depth, width and wear loss volume. The wear loss volume determination was carried out by calculating the area of the wear track profile using the trapezoid rule, followed by the calculation of the volume by using the Archard equation^23^.

To investigate the presence of cracks and White Etching Areas (WEAs), a ‘standard’ serial sectioning method was adopted and the sections were made at 50 μm intervals. The sectioning involved the use of ATM-OPAL 410 for mounting samples in Bakelite prior to grinding/polishing with a Struers Tegra automatic polisher with interchangeable polishing platens. A Vickers indent was used to indicate the depth removed between every section. To reveal the microstructure, cross-sections were etched in Nital (98% Methanol, 2% Nitric Acid; COSHH form Attached) for 15 seconds according to ASM guidelines^24^. For each raceway, the location of sections was chosen as the region of greatest flaked/cracked areas.

**Figure 2:**
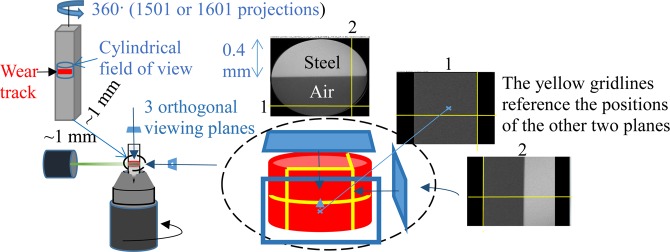
Micro-CT scan set-up.

The micro-CT scanning has been carried out with a Xradia Versa CT scanner using the settings listed in Table 2. A schematic of the micro-CT scan set-up is presented in Fig. 2.

**Table 2:** Micro-CT scan settings.

Shorter scans ~17 hours	Voltage/Power	160 kV/9 W
	Projections	1501
	Exposure	30 s
	Voxel size	0.79 µm
Longer scans ~60 hours	Voltage/Power	160 kV/9 W
	Projections	1601
	Exposure	120 s
	Voxel size	0.79 µm

The use of micro-CT as a technique for visualising the sub-surface damage was a challenging process for a number of reasons. The Z value of steel meant that long exposure times were necessary to achieve adequate quality to contrast ratios meaning very long scan times. Sometimes, scans would have to be run twice due to excessive noise in the first or the field of view not aligned with the wear track. For raceway specimens, each identified scan location was first sliced using ceramic coated cutting discs in the Mecatone T210 and manual grinders ground the specimen to suitable dimensions. For ball specimens a bespoke stand was manufactured to hold the ball for the duration of a scan. The region of interest would be positioned to sit at the highest point when in the stand.

XPS analysis of the disc tribofilms was carried out on a Thermo Scientific K-Alpha spectrometer (East Grinstead, UK) equipped with a 1486.6 eV microfocused monochromatic Al (Kα) X-ray source. The spot was ellipse shaped (200 × 400 μm^2^) and the pass energy was 200 eV for the wide (survey) spectra and 40 eV for the high resolution regions (narrow spectra). The instrument has an argon gun used to clean the samples by sputtering. A raster size of 1 × 2 mm^2^ and the Ar gas cluster ion beam (GCIB) mode at 6 keV with 1000 atoms for 30 s were used.

The sample tested in the Air/H_2_/Argon will be referred to as Air/H_2_/Argon specimen in the ‘Results and Discussion’ section of this paper.

## Results and Discussion

### Hydrogen content and fatigue life

RCF tests lubricated with PAO32 base oil were performed to failure of the bearing in the conditions described in Table 1.

The fatigue life of individual tests and the specimens that failed are detailed in Table 3 along with the total amount of hydrogen released from the specimens (disk and ball) during the TDS analysis. When quantifying the hydrogen released from the specimens, all hydrogen containing species (including the water) have been considered, as discussed in detail in one of our previous studies^19^.

**Table 3:** Hydrogen content and Fatigue Life.

Atm./Lube	Content of Hydrogen ppm		Failure	Fatigue Life Cycles
	Disc	Ball		
Air/PAO32	0.322	0.171	On disc	11,830,000
Ar/PAO32	0.283	0.226	On disc	9,310,000
H_2_/PAO32	0.406	0.672	On ball	6,600,600

The TDS results show the content of hydrogen in the ball and disc specimens follows the order H_2_ > Air > Argon with the only exception of the average content of hydrogen in ball for Ar being slightly larger than the average measured in the Air atmosphere. This is in line with the theoretical predictions. The sources of atomic hydrogen in the testing conditions employed were the hydrocarbon oil and the water solubilized in it (in all environments), the air water vapour (only in Air environment) and the hydrogen gas (only in Hydrogen environment). Therefore, the amount of hydrogen in specimens was expected to follow the order H_2_ > Air > Argon.

In line with the hydrogen content results, the fatigue life of specimens follows the opposite trend as it was longest in Air and shortest in Hydrogen. These results basically follow those previously reported by Tanaka^7,8^ in studies which investigated the statistical life of the bearing in the three environments. As seen in Table 3, regardless of environmental gas, the fatigue life decreases with the increase of the hydrogen content in specimens. However, fatigue life is also affected by other factors such as surface damage mechanisms, wear and degradation of lubricant (exacerbated in high temperature testing conditions).

As shown in Table 3, distinctively in Hydrogen, the failure took place on the ball, which also contained the largest amount of hydrogen of all specimens (4 and 3 times more hydrogen than the Air and Argon balls). For specimens tested in Air and Argon the hydrogen content was higher in discs than balls and the failure occurred on the discs. This situation was reported to be a general trend for RCF testing^1^ and was explained by two factors. The contact time between the race and ball wear tracks is higher for the race than for the ball because the balls are free to roll and the contact is mobile while the race/disc is continuously subjected to stress by the contact with the six balls. Also, in the case of the balls, the TDS results can be influenced by their volume and spherical shape, as the hydrogen content is likely to be higher at the surface than at the centre of the ball but is calculated as ppm per ball mass.

### Wear track analysis

**Figure 3:**
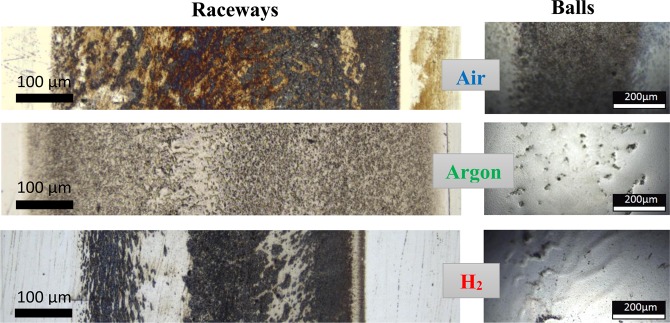
Optical light micrographs of the wear tracks of raceways and balls.

Optical light micrographs of the wear tracks on raceways and balls presented in Fig. 3 show significant differences between the three environments. The width of the wear tracks on raceways decrease in the order H_2_ > Air > Argon. The appearance of the tribofilms is significantly distinct. The wear tracks of Air specimens (raceway and ball) are uniformly covered by a dark tribofilm. The race wear track shows large areas of brown discoloration that could indicate rust, a feature not observed on the Argon and H_2_ raceway wear tracks. The H_2_ tribofilm has dark stripes in the rolling direction while the Argon wear track is uniformly covered by grey specks. The Argon and H_2_ wear track on the balls are not covered by tribofilms and show different types of damage. The Argon ball shows micropitting while the H_2_ ball displays distinctive protruding streaks running along the wear track which could act as precursors to spalling.

The presence of the tribofilms on the wear track (Fig. 3) has significant implications on the amount of hydrogen generated and permeated into specimens (Table 3). Intriguing, the TDS results showed the Ar specimens contained more atomic hydrogen than the Air ones despite the higher potential of the Air environment to generate atomic hydrogen due to its humidity.

In Air, the average content of hydrogen (disc and ball) was the lowest and the fatigue life was the longest. This indicates the ability of tribofilms on the Air specimens to stop the atomic hydrogen being generated and permeating them.

Despite the H_2_ environment posing a significantly increased (as detailed earlier) potential to generate atomic hydrogen, the tribofilm formed on the disc wear track led to a relatively small increase in hydrogen content as compared to the Air and Argon discs. In the case of the ball, the fact that the tribofilm did not form and the type of the damage generated on the ball wear track along with the high hydrogen content led to the premature ball failure (after only half or two thirds of the cycles performed in Air or Argon). This emphasizes the importance of the tribofilm in preventing the wear and hydrogen damage.

### Wear mechanisms

**Figure 4:**
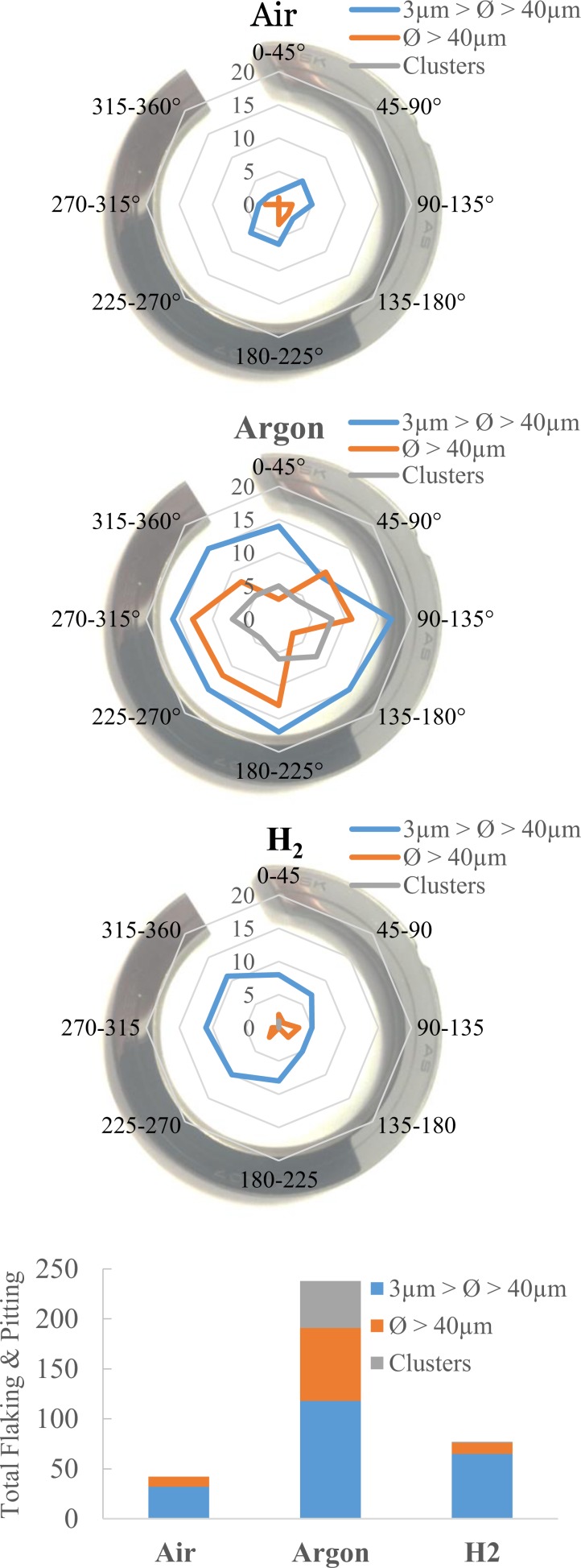
Wear track damage: total flaking and pitting; (**a**–**c**) are schematics of the wear tracks as received and locations where defects are found, (**d**) histogram showing the direct type of damage observed under the three test conditions.

Optical investigation of pitting/flaking represented cumulatively in Fig. 4, shows the Argon atmosphere raceway exhibits the most defects. This is attributed to the increased surface distress, a surface initiated fatigue at an asperity level corresponding to high surface tractions. On the other hand, in the oxygen abundant air environment the raceways were comparably free of flaking.

Surface distress can also be related to the more common occurrences of flaking/spalling with approximate diameter >40 μm and groups of 4+ defects (clusters) in the specimens as added debris in the lubricant from the micro-pits that then facilitate and accelerate other failure modes through increased abrasive wear^25^.

H_2_ and Air encouraged a different wear mechanism involving smaller wear particles, explaining the observations from optical microscopy showing that the micropitting on the raceway though frequent, its diameter was rarely above 15 μm. This mechanism can be termed mild material removal (MMR) and is characteristic of enhanced lubrication conditions, said to compete with surface distress during the running-in stage. This explains the lack of large flaking defects as the surface layers of the material are continually removed, eradicating the propagation of micro-cracking caused by surface distress^25,26^.

**Figure 5:**
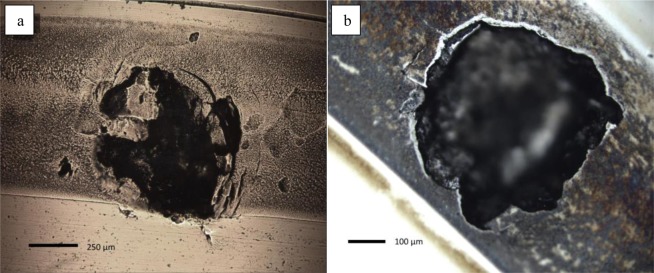
Optical micrographs of raceway failure from spalling in (**a**) Argon (**b**) Air.

Figure 4 goes further to demonstrate the difference in flaking exhibited by specimens tested in Argon compared with Air, however their eventual failure was somewhat similar as shown by the large spalls in Fig. 5. The lack of flaking on the wear track in Air suggests that failure was primarily due to sub-surface cracks, whereas the Argon specimens spall could have been surface initiated from continual ploughing of flaked areas, meaning friction may have been a larger limiting factor in service life^27^.

**Figure 6:**
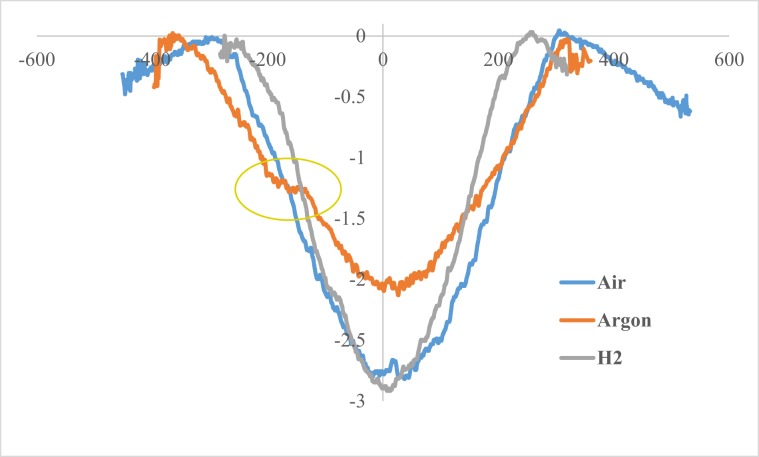
Profiles of race wear tracks.

Figure 6 compares the typical 2D profiles of the Air, Argon and Hydrogen raceways as measured with optical profilometry. As previously reported^19^, during high load testing, the edges of the wear track were raised slightly to an almost similar degree in all specimens due to plastic deformation. The height of the side ridges/furrows only changes if there is additional pile-up due to wear. The Air atmosphere resulted in a narrower deeper wear track than the specimen tested in Argon which demonstrated a shallower and wider channel. This feature is accompanied by the increased likelihood of ‘w’ shape wear grooves in the Argon raceway by a subtle lip across the measurement region. Kingsbury accredited a ‘w’ shape to pivot slip and relatively small drag slip in the wear track^28,29^. The amount of wear and the ‘w’ shape were related to the poor ability of the lubricant to readily form an insulating film of friction polymer on the wear track and the extensive generation of wear debris^29^.

**Figure 7:**
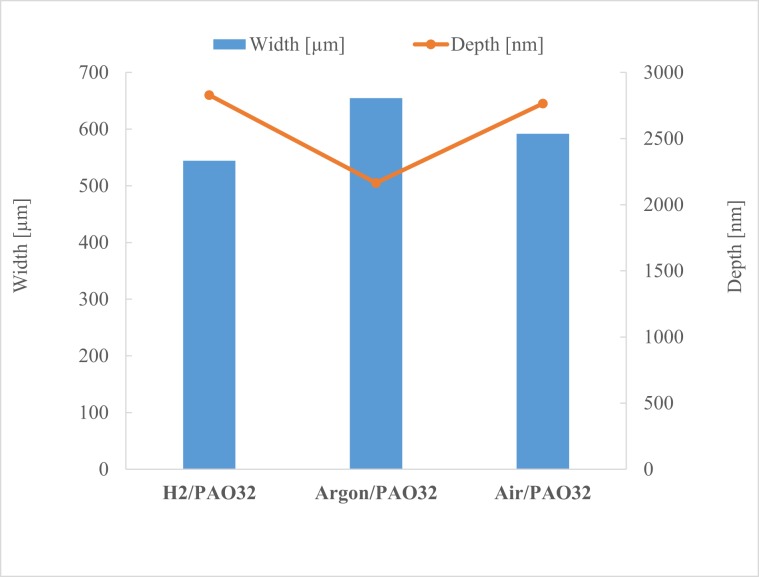
Wear track mean width and depth.

**Figure 8:**
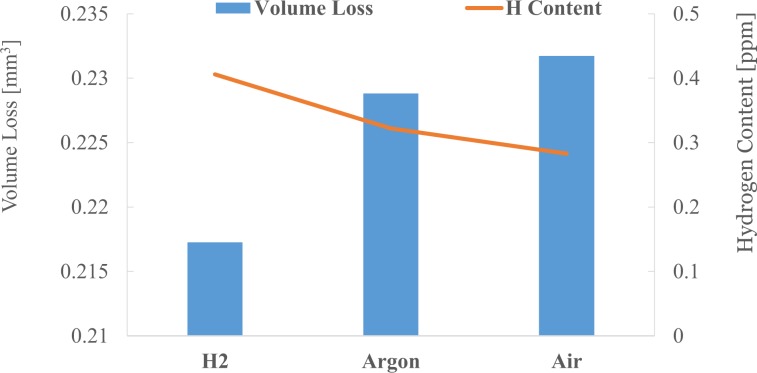
Wear volume loss of the race wear track and the Hydrogen content.

Figure 7 presents the depth and width of the wear scars. While the depth decreased in the order H_2_ > Air > Ar, the width and the calculated wear loss (Fig. 8) increased in that order (H_2_ < Air < Ar). This is also the order in which the content of hydrogen in the discs decreased (Fig. 8).

**Figure 9:**
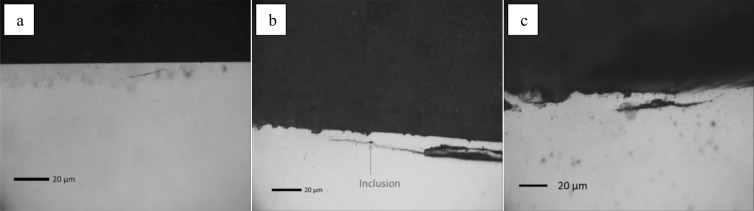
Serial sectioning of the race specimens revealing subsurface cracks in (**a**) Air, (**b**) Argon, (**c**) H_2_.

The sectioning process revealed a small number of subsurface cracks in races as seen in Fig. 9(a–c).

The areas of the specimens subject to CT scans were chosen primarily based on the location of large flaking clusters indicating positions where sub-surface activity had taken place.

**Figure 10:**
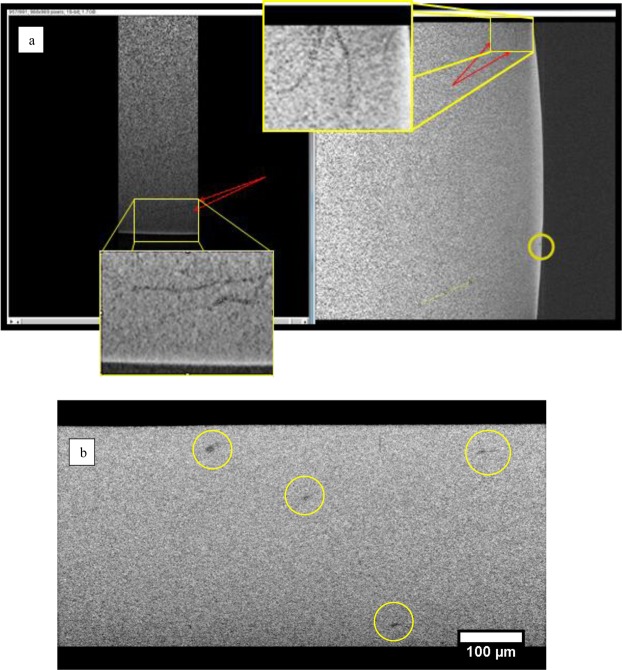
µ-CT showing (**a**). Base view (left) and Right view (right) exhibiting a multiple pathed crack propagating parallel to the surface of the H_2_ ball, (**b**). Pores in the H_2_ race wear track subsurface.

The screen shots from the reconstructed CT-scans in Fig. 10 demonstrate the results of the preliminary scans to test the ZEISS VRSA capabilities for capturing subsurface damage. The CT performed on the H_2_ ball bearing that failed (Table 2) was promising partially due to the contrast to noise ratio increasing towards the edge of the ball where transmissivity peaked due to decreased ‘thickness’, however deeper cracks were comparably unperceivable. Figure 10(a) demonstrates a multiple path crack visible in the ‘Base view’ of the scan as separate cracks which can be seen in the ‘Right view’ to converge to a single crack. The feature circled in Fig. 10(a) shows a small axial crack before surface contact occurs (depth 3 μm). CT also revealed pores in the H_2_ race track (Fig. 10(b)). The detail exposed by the CT is similar to that observed by optical microscopy and demonstrates that CT scanning can be a useful technique in revealing subsurface damage in RCF tests.

### Chemical analysis of tribofilms present

**Figure 11:**
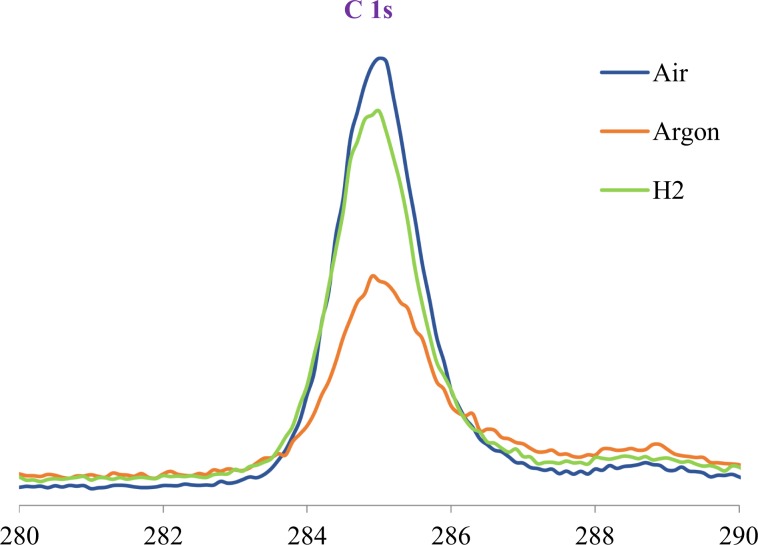
C 1s spectra at the surface of the race wear track.

**Figure 12:**
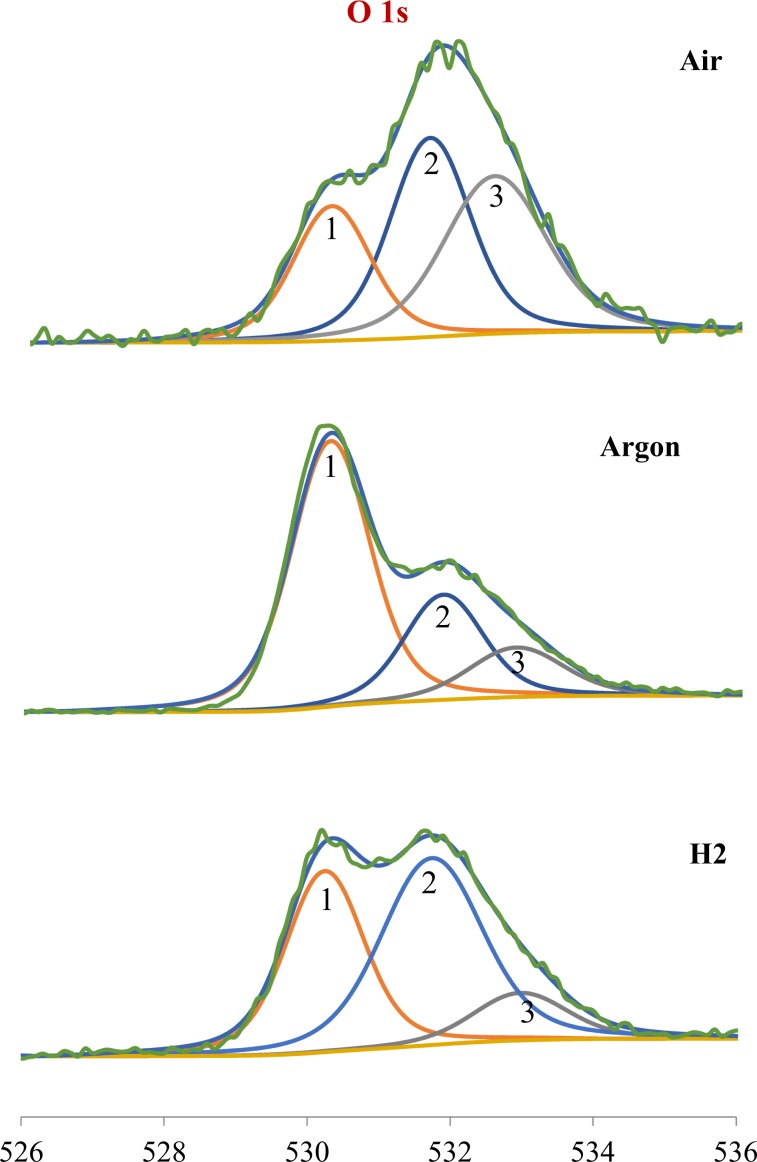
O 1s spectra at the surface of the race wear track.

To investigate the nature of the tribofilm formed on the wear track during the fatigue life tests and to measure the elemental composition and chemical state of elements, XPS analysis was carried out. The signal values have been verified against literature data^30^. The wear track of specimens exhibited major signals for iron in both its oxide (708.9 eV and 718 eV) and elemental state (706.6 eV), oxygen in the form of Fe-oxides (530.5 eV) and C=O bonds (531.7 eV) and carbon as C–C/C–H (285 eV) and C=O (289 eV). The C 1s and O 1s signals measured at the surface of the specimens are shown in Figs. 11 and 12. C 1s analysis on the surface of tribofilms (Fig. 11) revealed a major peak, C–C (284.8 eV) that is almost twice as large in Air and H_2_ as in Argon which indicates the presence of a significant carbonaceous accumulation on the surface of these tribofilms. There is also a small C=O (289 eV) peak for all specimens.

The surface O 1s (Fig. 12) signal can be deconvoluted into three peaks: **1** (530.5 eV) is attributed to Fe-oxides, **2** (531.7 eV) to C=O bonds and **3** (532.1 eV) to contamination. The Ar tribofilm shows a predominant peak **1** and a small peak **2** indicating that its surface is covered mainly by iron oxides. For the Air specimen all three peaks are prominent but peak **2** is larger than for the other two specimens. The H_2_ tribofilm shows significant and almost equal peaks **1** and **2**. Peak **3** (indicating contamination) is small in Argon and H_2_ but large in Air.

**Figure 13:**
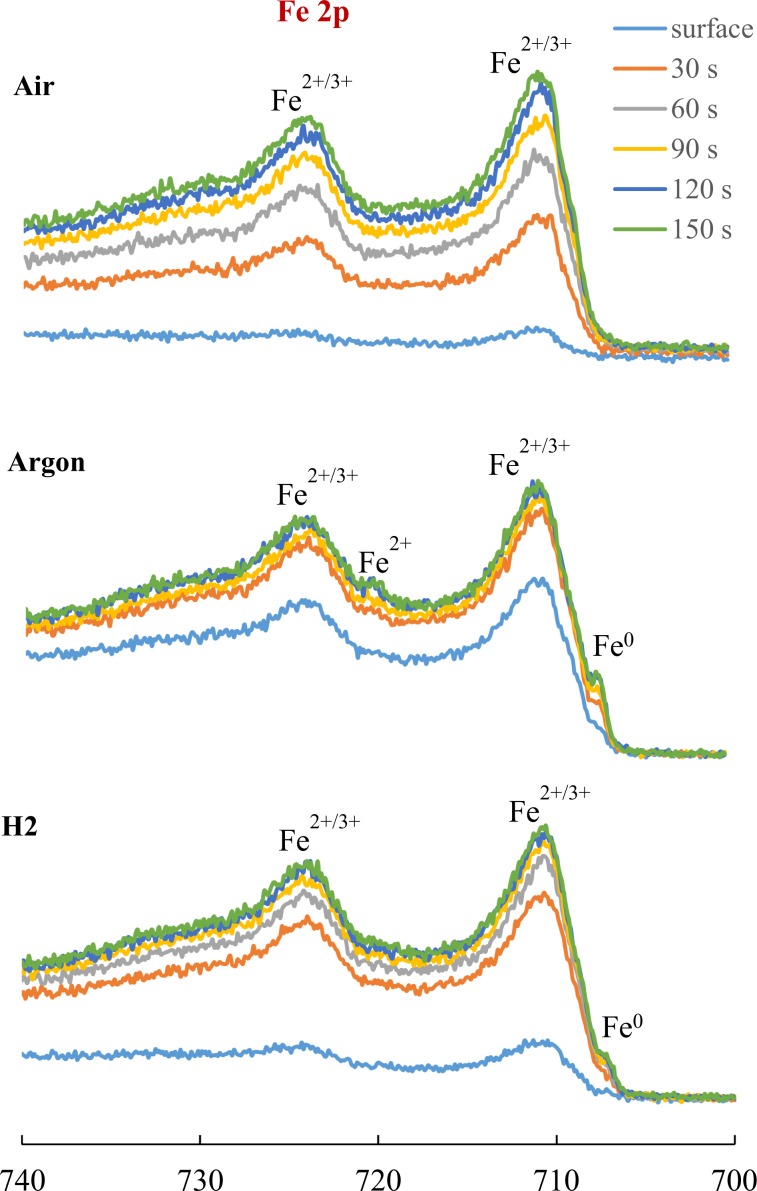
XPS Depth profile spectra for Fe 2p.

The depth profile spectra for Fe 2p recorded during the first 150 s of sputtering are shown in Fig. 13. In all specimens iron is mostly found in its Fe^2+/3+^ oxide form (710 eV and 724 eV). However, the signals recorded at the surface of Air and H_2_ specimens are minimal. After 30 s of sputtering the Fe^0^ signal started to be recorded for both Ar and H_2_ specimens while Ar also showed the Fe^2+^ peak (718 eV). No significant changes in the signal intensities are recorded for Ar and H_2_ specimens after 30 s of sputtering of the former and 60 s of the latter which indicates the carbon layer was thinner on these than on the Air specimen where the intensities continue to increase through the 150 s of sputtering time.

**Figure 14:**
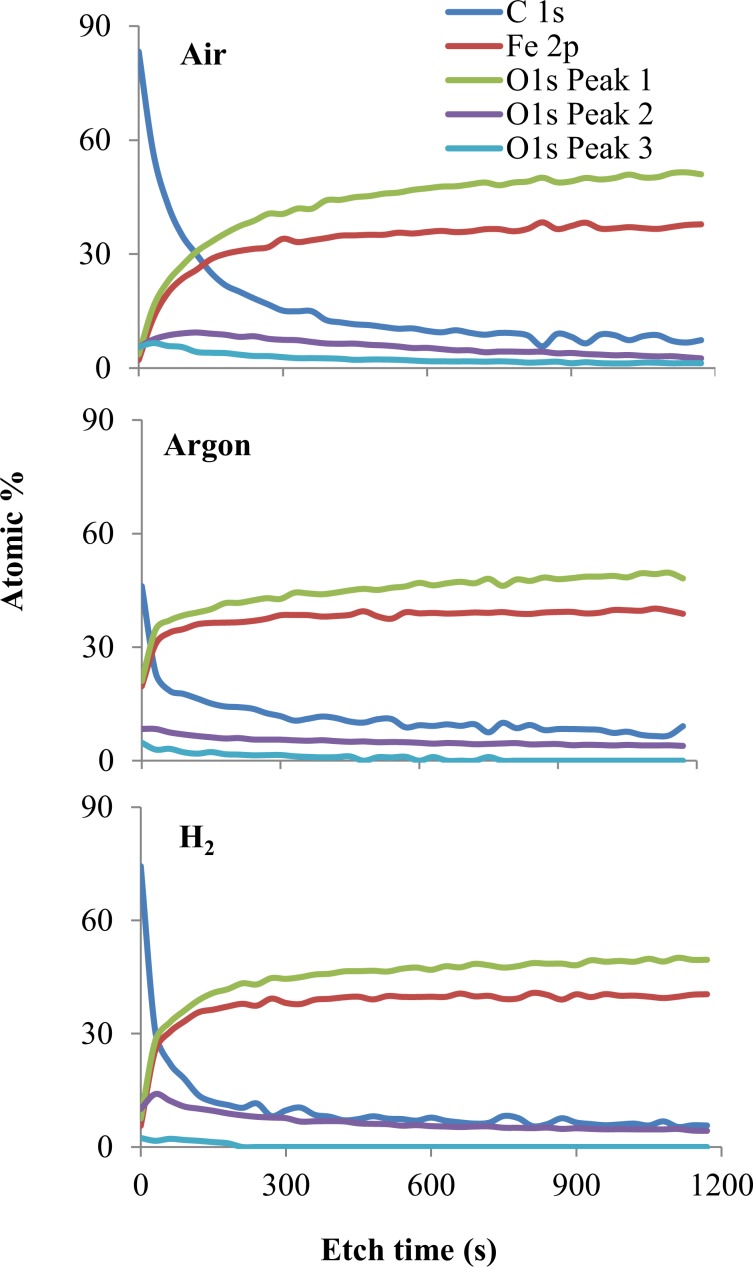
XPS atomic% depth profiles for C 1s, Fe 2p and O 1s peaks.

A comparison of the XPS intensities of the three specimens is depicted in Fig. 14 in the form of atomic% depth profiles for C 1s, Fe 2p and O 1s signals. At the surface, mainly carbon was detected but the amount is considerably higher for Air and H_2_ specimens than Ar. The C 1s curve decreased more slowly with sputtering for Air which confirms the carbon containing layer is thicker in Air than in H_2_ and Argon. These layers were formed from the degradation of the oil. Underneath this, the tribofilm contains mainly iron, oxygen and some carbon. The Air specimen showed the largest iron oxide content.

This chemical composition of tribofilms generated by base oils in Air, Argon and H_2_ can explain the morphology of the wear tracks. The C 1s, Fe 2p and O 1s depth sputtering analysis indicate that the tribofilms are mainly formed of carbon, iron and oxygen. The significant difference is that the Air and H_2_ films contain more carbon (especially on the surface) and iron oxides than Argon. These results are directly related to the different nature of the test environments. The oxygen abundant Air atmosphere favours oxypolymerization and solidification of the lubricant hydrocarbon. It has been shown that the presence of nascent metal surfaces in the system acts as a catalyst to further increase the rate of oxypolymerization^31,32^. However, in the inert Argon atmosphere this process is not achievable. Both the Argon and Hydrogen gas were of high purity which implies that the oxide layer formed on the Argon and Hydrogen wear tracks was due to the air and water solubilized in the lubricant. It is also possible that the iron oxide layer on the Argon wear track formed after the RCF test when the specimen was exposed to Air as the very thin carbonaceous surface layer could make the Argon wear track more prone to oxidation.

In the case of the H_2_ specimen, the dark tribofilm visualized by optical microscopy and shown by the XPS analysis to be a carbonaceous layer has a different provenance than the Air carbon rich tribofilm. If the effect of H_2_ environment on steel tribological parts has been extensively researched, its influence on the hydrocarbon lubricant has been largely ignored. Literature on catalysis reports that hydrogenolysis, a type of catalytic hydrocracking which involves lysing of carbon-carbon single bonds followed by hydrogenation with hydrogen gas, takes place on metallic catalysts in the presence of H_2_ gas under pressure and high temperatures^33^. Iron films can act as catalysts for hydrogenolysis but they require higher temperatures than other metal catalysts. This has been attributed to the ease with which iron dissociatively and irreversibly adsorbs hydrocarbons^34^. At temperatures sufficiently high for hydrogenolysis to occur it has been found that the rate of the reaction displays a positive dependence on hydrogen pressure (i.e. standard hydrogenolysis conditions of n-butane are P~30 MPa, T > 113 °C) and it can be a consequence of the fact that the hydrocarbon radicals are tightly held on an iron surface. The reactivity for hydrogenolysis increases as the number of carbon atoms in the molecule increases. Arrhenius plots used to analyse the effect of temperature on the rates of chemical reactions show that the temperature of the maximum reaction rate decreased with the increase in the number of atoms in the hydrocarbon reactant^35^. The main effect of hydrogenolysis is the cleavage of the hydrocarbon chains which leads to a mixture with a lower chain length distribution. It also results in an accumulation of carbonaceous material on the surface of the catalyst^35^. Thus, the operating conditions of the EHD tribological contact (pressures in the range 1–5 GPa, T > 120 °C, up to 10% slide roll ratio) exposed to hydrogen atmosphere can favour hydrogenolysis. In the case of a hydrocarbon lubricated tribological contact hydrogenolysis may lead to the formation of a carbonaceous tribofilm over the nascent surfaces (wear track) which act as a catalyst. It has been shown that tribofilm formation on the wear track can attenuate the generation and permeation of atomic hydrogen and therefore prevent HE of bearing steel^1,19^. The cleavage of the hydrocarbon chains could result in a decrease in lubricant viscosity over time. This effect was not investigated in this study, but it should be monitored and controlled during future tests.

The carbonaceous layer on the wear track results from the environment effect on the lubricant. Both Air and Hydrogen atmospheres can generate it, but each may follow distinctly different chemical mechanisms, namely oxypolymerization in the case of the oxygen rich Air and hydrogenolysis for Hydrogen. Both processes rely on the nascent steel surfaces generated through wear to catalyse the hydrocarbon decomposition reactions. They are assisted by the high pressure and temperature conditions along with the presence of the oxygen in Air environment, or the Hydrogen gas in Hydrogen atmosphere. In the case of Argon, the friction, temperature and pressure proved not enough to degrade the lubricant and generate a useful (thick and uniform) carbonaceous tribofilm. The lack of it explains why this wear track exhibited the most defects (pitting/flaking) and larger pivot and drag slip. Despite the Argon atmosphere having a lower potential than Air to generate atomic hydrogen (contains no water) in bearing lubrication, the fatigue life is lower and hydrogen content is higher.

## Conclusions

It has been shown that hydrocarbon lubricants can control hydrogen embrittlement by influencing the generation and characteristics of the wear track tribofilm and therefore the wear mechanism. By controlling wear, the tribofilms led to smoother wear tracks and reduced hydrogen permeation into the bearing steel during the testing. The mechanisms through which these effects were achieved are: (1) build-up of a tribofilm through oxide formation and lubricant degradation on the wear track, which obstructs the formation of nascent metal sites that catalyse the decomposition of oil/water/hydrogen gas molecules and the generation of atomic hydrogen; (2) the tribofilm functions as a physical barrier for hydrogen permeation; (3) hydrogen contributes to the generation of the tribofilm by redox reactions (i.e. reducing the chemical state of iron from Fe^2+/3+^ to Fe^0^). Additionally, a previous study^19^ showed that tribofilms reduce the total amount of water (in the steel, at the surface, in bulk -adsorbed via defects) and arising from the reaction of hydrogen atoms with oxides), thus inhibiting rusting and extending the life of the steel.

The tribofilm consists of mainly solidified cracked or oxy-polymerized carbon species and iron as oxides and elemental forms and its thickness decreases in the order Air > H_2_ > Ar.

In the inert Argon atmosphere where no oxypolymerization or hydrogenolysis of the hydrocarbon lubricant takes place, the tribofilm was thinner and contained less carbon and iron oxide. This led to extensive surface initiated spalling and pitting and therefore reduced service life.

The impact of the tribofilm on the service life extension of steel becomes especially important in H_2_ environment where atomic hydrogen is generated from both the environment and lubricant and failure rates are high. The tribofilm generated in H_2_ atmosphere has a composition and thickness comparable to the Air film. This could result from hydrogenolysis of the hydrocarbon lubricant, promoted by the presence of Hydrogen gas and nascent iron sites on the wear track which act as a reaction catalyst. The chemical composition of the Air and H_2_ tribofilms can explain both the reduced surface damage (flaking and pitting) on the races and the failure on the ball for the tests in Hydrogen environment.

It was found that the use of Micro-CT as a non-destructive technique for investigating damage gives a reasonable crack depiction and is also capable of revealing 3D crack morphology. Replacing µ-CT with a monochoromatic synchrotron would help to avoid the problems associated with the high density of steel (such as noise) and the long duration of the scanning as it offers more coherent X-ray sources and much faster imaging.
